# The genus *Massalongia* (lichenised ascomycetae) in the Southern Hemisphere

**DOI:** 10.3897/mycokeys.60.37725

**Published:** 2019-12-04

**Authors:** Per M. Jørgensen, Heidi L. Andersen, Arve Elvebakk

**Affiliations:** 1 Dept. of Natural History, University Museum of Bergen, Allégt. 41, N-5017, Bergen, Norway University Museum of Bergen Bergen Norway; 2 Tromsø University Museum, University of Tromsø – the Arctic University of Norway, PO Box 5060 Langnes, N-9037, Tromsø, Norway University of Tromsø – the Arctic University of Norway Tromsø Norway

**Keywords:** Peltigerales, Massalongiaceae, phylogeny, taxonomy, South Hemisphere

## Abstract

The species of *Massalongia* recorded and described from the Southern Hemisphere are revised and it is shown that only one is present; *M. patagonica* which is widespread, with populations in Australia and New Zealand that differ from the South American populations, but at present best regarded as part of the variation of that species. Records from this hemisphere of all other species placed in the genus are incorrect. The type species, *M. carnosa*, is restricted to the Northern Hemisphere. Two species, *M. antarctica* and *M. novozelandica* cannot be identified precisely due to lack of sufficient type material and with the types as the only collections known of these, but none belongs in *Massalongia* according to available data. *Massalongia griseolobata* (from Gough Isl.) is shown here to belong in the Pannariaceae and is part of the parmelielloid clade. *M. intricata* (from South Georgia) and *M. olechiana* (from South Shetland) have both recently been correctly transferred to the genus *Steinera* in the Arctomiaceae.

## Introduction

The genus *Massalongia* was described by Körber (1855), based on the species *Lichen carnosus* described by J. Dickson in 1790 on material collected in Scotland, but later often called *Pannaria muscorum* (Ach.) Duby, an illegitimate, superfluous name. This reflects the difficulties which the early lichenologists had in classifying the species. Molecular studies (Wedin et al. 2007; Muggia et al. 2011) have shown that *Massalongia* does not belong in the Pannariaceae, but is best placed in a family of its own, Massalongiaceae, in the Peltigerales. There is, however, only one major study of the species and their variation, made by Henssen (1963) covering North America. She accepts two species; the widespread, variable *M. carnosa* and the nearly crustose, microphylline, local Californian endemic *M. microphyllizans* (Hasse ex Nyl.) Henssen. Jørgensen (2000), whilst revising the Pannariaceae, had studied the type of *Massalongia fauriei* (Hue) Zahlbr. and found the poor type (the only material existing) to belong in *Fuscopannaria leucophaea* s.lat., now transferred to the genus *Vahliella* (Jørgensen 2008; Wedin et al. 2010). An additional Asian species has been recorded from the Philippines based on Rehm (1916). This is, however, based on a misunderstanding of *Massalongiella imperatae* Rehm., which is a non-lichenised ascomycete as originally described. In Europe, Harmand recognised a variety which Gyelnik (1940), in his notorious treatment of the Pannariaceae in Rabenhorst’s Kryptogamenflora, raised to species rank as *Massalongia meizospora*, only representing a form with somewhat longer, 3-septate spores. In addition, he established a new *Massalongia rabenhorstiana*, the type of which has disappeared. It is most certainly only a synonym of *M. carnosa*, still the only species on the Northern Hemisphere in addition to *M. microphyllizans*, a species in need of a phylogenetic study.

The situation in the Southern Hemisphere is different, though it took a long time before any species in the genus was recognised. Zahlbruckner (1917) was the first when he recorded *Massalongia carnosa* from the Falkland Islands, followed by Lamb (1958) who recorded it from Patagonia. Later, it was mentioned from several regions in the Southern Hemisphere (Smith and Corner 1973; Lindsay 1974; Galloway 1985; Redon 1985; Jørgensen 1986; Jørgensen and Elix 1988; Øvstedal and Smith 2001). In addition, several new species were described from the Southern Hemisphere; *M. antarctica* Dodge (from the Antarctic Peninsula, Dodge 1971), *M. novozelandica* Dodge (from subantarctic New Zealand, Dodge 1971), *M. griseolobata* Øvstedal (from Gough Isl., Øvstedal and Gremmen 2010), *M. intricata* Øvstedal (from South Georgia, Øvstedal and Smith 2001) and *M. olechiana* Alstrup & Søchting (from South Shetland, Alstrup and Søchting 2011).

During fieldwork in Chile in 2015, one of the authors (A.E.) discovered a strange *Pannaria*-like lichen which, on closer inspection, proved to be a *Massalongia* with some differences from *M. carnosa*, as known by us from Norway. However, since this is a variable species, we felt that a more detailed study, including molecular screening, would be useful. This being done and the distinction of this material proven, we found it necessary to check on the surprisingly high number of species of *Massalongia* described from the Southern Hemisphere. This proved to be time-consuming and complicated, since it was difficult to get hold of suitable material and, when molecularly checked, often not giving clear results and involving quite unrelated lichen families. Fortunately, Ertz et al. (2017) solved some of our problems and, eventually, this new species was named by Kitaura and Lorenz in Liu et al. (2018). However, our project contains more data than their work includes and we present these here in an attempt to give full clarification of the taxonomic situation for the genus in the Southern Hemisphere. Some additional phylogenetic data were also added on the genus in the Northern Hemisphere.

## Material and methods

### The specimens

Specimens of *Massalongia* were obtained from various herbaria for phylogenetic analyses, see Tables 1 and 2. In addition, we microscopically studied material from the following herbaria: BAA, BG, BM, C, CANB, CANL, CHR, F, FH, H, MSC, NY, TROM and UPS. A total of 130 ascospores from collections from both hemispheres were drawn in detail and measured for comparison.

**Table 1.** List of specimens used for phylogenetic analyses of the broad analysis of the *Massalongiaceae* and *Pannariaceae*, with vouchers and accession numbers from GenBank. Bold accession numbers are new in this study.

Species and ID	Voucher	mtSSU	RPB1
*Austrella arachnoidea*	Jørgensen 8200 (BG)	KC608054	KC608108
*Collema furfuraceum*	Wedin 6187 (BM)	AY340488	GQ259048
*Collema nigrescens*	Wedin 7046 (UPS)	GQ259020	GQ259049
*Collema parvum*	Nordin 5500 (UPS)	GQ259021	GQ259050
*Degelia duplomarginata*	Wedin 8023 (S)	KC608058	–
*Degelia durietzii*	Elvebakk 02-296 (TROM)	KC608059	–
*Degelia gayana*	Wedin 6112 (UPS)	AY652619	–
*Degeliella rosulata*	Galloway 840b (BG)	KC608063	–
*Degeliella versicolor*	Galloway 840a (BG)	KC608064	–
*Erioderma pedicellatum*	mrSSU: MacPitcher s.n. 2007 (BG-L85909), RPB1: MacPitcher s.n. 2007 (BG-L85911)	KC608065	KC608110
*Erioderma verruculosum*	AFTOL-ID 337	DQ972990	DQ973062
*Fuscoderma applanatum*	Tibell 19076 (BG)	KC608112	KC608066
*Fuscopannaria pacifica*	Tønsberg 29359 (BG)	KC608074	KC608118
*Fuscopannaria praetermissa*	mrSSU: Tønsberg 36838 (BG) RPB1: Wedin 7671 (UPS)	KC608075	GQ259056
*Joergensenia cephalodina*	Passo 269 (BCRU 4895)	EU885330	–
*Lecidea fuscoatra*	Wedin 6860 (UPS)	AY756401	AY756408
*Leciophysma furfurascens*	Nordin 5695 (UPS)	GQ259028	GQ259058
*Leioderma erythrocarpum*	Schumm and Frahm s.n. 2009 (BG, dupl of hb. Schumm 15583)	KC608078	–
*Leioderma pycnophorum*	Wedin 8013 (S)	GQ259031	GQ259059
*Leptochidium albociliatum*	Tønsberg 29087 (BG)	DQ900632	GQ259060
	Muggia TSB38886	JF938191	–
	Spribille 20997 (COLO)	JF938193	–
*Lobaria pulmonaria*	mrSSU: Wedin 6167 (UPS) RPB1: Wedin 5092(UPS)	AY340503	GQ259068
*Lobaria scrobiculata*	AFTOL-ID 128	AY584621	DQ883736
*Massalongia carnosa*	Tønsberg 44267 (BG)	**MN708314**	**MN714653**
	Tønsberg 45410 (BG)	**MN708315**	**MN714654**
	Johnsen L-86694 (BG)	**MN708316**	–
	Ezhkin 1289 (SAK)	**MN708317**	**MN714655**
	Spribille 22021 (COLO)	JF938205	–
	Haikonen 20961 (H)	EU558817	–
	Hermansson 8916 (UPS)	AY340509	GQ259071
	Spribille 21565 (COLO)	JF938204	–
*Massalongia patagonica*	Elvebakk 99:775 (TROM)	**MN708318**	**MN714656**
	Elvebakk 15:033 (SGO)	**MN708319**	**MN714657**
	Buck 60287 (NY)	**MN708320**	**MN714658**
	Gremmen K-789 (BG)	**MN708321**	**MN714659**
	Galloway 5616 (CHR)	**MN708322**	**MN714660**
	Galloway s.n. (CHR)	**MN708323**	**MN714661**
	Kitaura 4181 (CGMS)	MG243608	–
	Kitaura 4188 (CGMS)	MG243607	–
	Kitaura 4168 (CGMS)	MG243609	–
*Massalongia griseolobata*	Gremmen 2006-91 (BG)	**MN708324**	–
*Nephroma parile*	Wedin 6169 (UPS)	AY340512	GQ259072
*Pannaria hookeri*	Jørgensen s.n. (BG)	KC608083	KC608124
*Pannaria immixta*	Elvebakk 02-352b (BG)	KC608084	KC608125
*Pannaria rubiginella*	Thor 10050 (S)	GQ259037	GQ259074
*Parmeliella appalachensis*	Lendemer 578 and Smith (BG)	KC608090	–
*Parmeliella miradorensis*	Tønsberg 23053 (BG)	KC608094	KC608136
*Parmeliella nigrocincta*	Elvebakk 02-356 (BG)	KC608095	KC608137
*Parmeliella pannosa*	Ståhl s.n. 1999 (BG)	KC608096	–
*Parmeliella triptophylla*	Wedin 7037 (UPS)	AY652623	GQ259075
*Pectenia atlantica*	Lindblom and Blom L251 (BG)	KC608057	KC608109
*Pectenia cyanoloma*	Purvis, James and Smith 1995 (BM)	AY340491	GQ259052
*Pectenia plumbea*	AFTOL-ID 1046	DQ912300	DQ912374
*Placynthium nigrum*	Wedin 6778 (UPS)	AY340518	GQ259079
*Polychidium muscicola*	Obermayer 8547 (UPS)	DQ900634	GQ959080
	Spribille 26411 (KLGO)	JF938220	–
*Pseudocyphellaria aurata*	Purvis, James and Smith 7/5/1995 (BM)	AY340520	GQ259082
*Psoroma hypnorum*	Ihlen 453 (BG)	KC608100	KC608142
*Santessoniella arctophila*	Kristinsson s.n. (BG)	KC608104	KC608145
*Sticta fuliginosa*	Wedin 6078 (BM)	AY340529	GQ259089
*Vahliella carnifornica*	Tønsberg 26316 and Goward (BG)	HQ268594	HQ268593
*Vahliella leucophaea*	Wedin 6849 (UPS)	AY652621	GQ259090

**Table 2.** List of specimens used for phylogenetic analyses of the species delimitation in *Massalongia*, *M.* referring to *Massalongia*, *P.* to *Polychidium* and *L.* to *Leptochidium*, with vouchers and accession numbers from GenBank. Bold accession numbers are new in this study.

**Species and ID**	**Voucher**	**Area**	**ITS**	**LSU**	**mtSSU**	**RPB1**
*M. carnosa*	Tønsberg 44267 (BG)	USA: Alaska	**MN708327**	**MN708327**	**MN708314**	**MN714653**
	Tønsberg 45410 (BG)	USA: Alaska	**MN708328**	**MN708328**	**MN708315**	**MN714654**
	Johnsen L-86694 (BG)	Norway	**MN708329**	**MN708329**	**MN708316**	–
	Ezhkin 1289 (SAK)	East Russia	**MN708330**	**MN708330**	**MN708317**	**MN714655**
	Spribille 22021 (COLO)	USA: Montana	–	–	JF938205	–
	Hermansson 8916 (UPS)	Sweden	–	AY340554	AY340509	GQ259071
	Rui andTimdal 13267 (O)	Norway	MG243601	–	MG243611	–
	Hansen 1138 (COLO, H)	Greenland	MG243599	MG243615	MG243610	–
	Hansen 1057 (H)	Greenland	MG243603	MG243616	MG243612	–
	Türk 17280 (H)	Austria	MG243602	MG243614	MG243613	–
*M. patagonica*	Elvebakk 99:775 (TROM)	Chile	**MN708331**	**MN708331**	**MN708318**	**MN714656**
	Elvebakk 15:033 (SGO)	Chile	**MN708332**	**MN708332**	**MN708319**	**MN714657**
	Buck 60287 (NY)	Chile	**MN708333**	**MN708333**	**MN708320**	**MN714658**
	Gremmen K-789 (BG)	Australia	–	–	**MN708321**	**MN714659**
	Galloway 5616 (CHR)	New Zealand	**MN708334**	**MN708334**	**MN708322**	**MN714660**
	Galloway s.n. (CHR)	New Zealand	**MN708335**	**MN708335**	**MN708323**	**MN714661**
	Kitaura 4181 (CGMS)	Argentina	MG243604	MG243617	MG243608	–
	Kitaura 4188 (CGMS)	Argentina	MG243606	MG243619	MG243607	–
	Kitaura 4168 (CGMS)	Argentina	MG243605	MG243618	MG243609	–
*P. muscicola*	Obermayer 8547 (UPS)	Austria	–	DQ900647	DQ900634	GQ259080
*L. albociliatum*	Tønsberg 29087 (BG)	USA	–	DQ900644	DQ900632	GQ259060

### DNA extraction, Amplification and Sequencing

Total genomic DNA was extracted using DNeasy Plant Mini Kit (Qiagen). Four DNA markers where amplified; the mitochondrial small subunit rDNA (mtSSU rDNA: primers mrSSU1 and mrSSU3R (Zoller et al. 1999)), the internal transcribed spacer (ITS) and the large subunit (LSU) regions of the nuclear ribosomal RNA gene (primers ITS1f (Gardes and Bruns 1993), ITS4 (White et al. 1990), LSU155 and LSU362 (Döring et al. 2000), LSU635/LR3 and LSU1125/LR6 (Vilgalys and Hester 1990)) and the gene coding for the largest subunit of RNA polymerase II (RPB1: primers PRB1-BCR (Wedin et al. 2009), gRPB1-A (Stiller and Hall 1997) and fRPB1-C (Matheny et al. 2002)).

PCR reactions consisted of 1× GeneAmp PCR Buffer II (Applied Biosystems), 2.5 µM MgCl_2_ (Applied Biosystems), 20 µM dNTPs (Promega), 0.4 µM of each primer, 0.03 U AmpliTaq DNA Polymerase (Applied Biosystems), 2–5.0 µl of genomic DNA extract and distilled water to a total volume of 25 µl. PCR reactions were performed on a C1000 Touch thermal cycler (Bio-Rad Laboratories), with the following temperatures; initial denaturation at 94 °C for 4 min, followed by a 62–56 °C touchdown annealing for the first 6 cycles, ending with 30 cycles at 56 °C for 30 sec, polymerisation at 72 °C for 1 min 45 sec and a final elongation at 72 °C for 10 min.

Direct sequencing of PCR products was run with the PCR primers using a BigDye Terminator Cycle Sequencing kit (Applied Biosystems) on an ABI Prism 3700XL DNA analyser (Applied Biosystems) at the DNA Sequencing Facility (UiB), Norway. Sequences were assembled and edited using Geneious v.11.0.2 (Kearse et al. 2012).

Newly generated sequences with GenBank accession numbers are listed in Tables 1, 2, together with sequences downloaded from GenBank.

### Phylogenetic analyses

To align the sequences, MAFFT v7.309 (Katoh et al. 2002; Katoh and Standley 2013) implemented in Geneious v.11.0.2 (Kearse et al. 2012) was used with default settings, followed by manual adjustments. Suitable substitution models for the separate datasets were identified using MrAIC v.1.4.6 (Nylander 2004).

Two different datasets were analysed; one broad analysis of *Massalongiaceae* and *Pannariaceae* to test whether the included species is part of *Massalongia* (Table 1) and a second analysis for species delimitation within *Massalongia* (Table 2). For the broader dataset, mtSSU and RPB1 were concatenated, using *Lecidea fuscoatra* as outgroup and for the species delimitation in *Massalongia*, mtSSU, LSU, ITS and RPB1 was concatenated using *Polychidium muscicola* as outgroup.

Separate analyses of all genes and concatenated datasets were run as Bayesian MCMC searches using MrBayes v.3.2.1 (Ronquist and Huelsenbeck 2003) with default options; substitution model GTR+G+I, 10 million generations starting with a random tree, four simultaneous chains and using the default temperature of 0.2. Every 1000^th^ tree was saved. Phylogenetic trees were visualised using Geneious v. 11.0.2 (Kearse et al. 2012).

## Results

### Phylogeny

The two resulting concatenated datasets consisted for the broad analysis of *Massalongiaceae* and *Pannariaceae* of 63 taxa with 1435 characters, whereas for the species delimitation in *Massalongia*, of 21 taxa with 2983 characters (details in Table 3).

**Table 3.** List of numbers of characters, taxa and constant variables, from the two concatenated datasets.

**Dataset**	**Numbers of characters**	**Numbers of taxa**	**Number of constant characters**	**Number of variable characters**
Broad analysis of the *Massalongiaceae* and *Pannariaceae*	1435	63	724	711
Species delimitation in *Massalongia*	2983	32	2745	238

**Figure 1.**
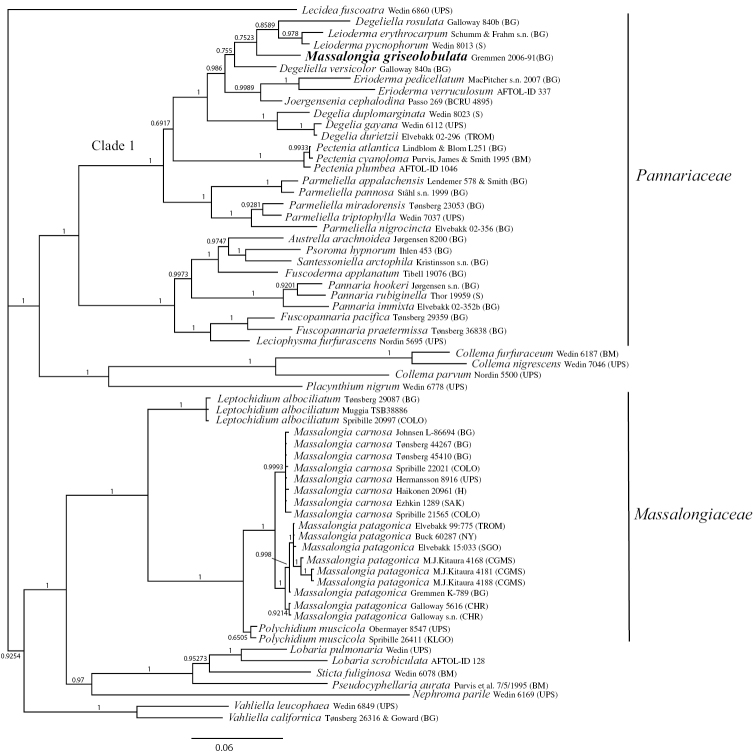
The phylogenetic tree of a concatenated broad dataset of the *Massalongiaceae* and *Pannariaceae*, resulting from Bayesian MCMC analyses.

The resulting phylogenetic consensus tree from the broad analysis of *Massalongiaceae* and *Pannariaceae* are given in Fig. 1. Both *Massalongia carnosa* and *M. patagonica* are with high support a part of the *Massalongiaceae*, together with *Polychidium muscicola* and *Leptochidium albociliatum*. *M. griseolobata* is a part of the Pannariaceae, in the “Parmelielloid” clade 1 from Ekman et al. (2014) with high support. Within this clade, *M. griseolobata* is a part of a supported group with no internal resolution, including *Degeliella rosulata*, *Degeliella versicolor*, *Leioderma*, *Erioderma* and *Joergensenia*.

**Figure 2.**
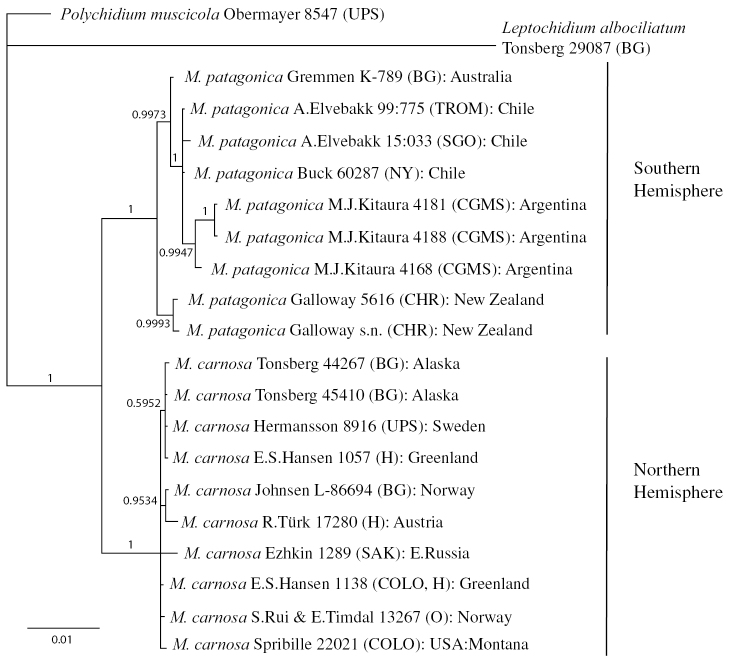
The phylogenetic tree of a concatenated dataset for species delimitation of *Massalongia*, resulting from Bayesian MCMC searches.

The resulting phylogenetic consensus tree from the species delimitation analysis of *Massalongia* is given in Fig. 2. *M. patagonica* from the Southern Hemisphere and *M. carnosa* from the Northern Hemisphere are nicely separated in two sister groups with high support.

The samples of *M. patagonica* from New Zealand are grouped in a separate subclade from the rest of the samples from Australia, Chile and Argentina. The phylogenetic tree indicates a high genetic variance within *M. patagonica* throughout the Southern Hemisphere, but further studies are necessary to evaluate these differences.

The samples from the Northern Hemisphere make a monophyletic clade with little variation between the samples and a sample from Sweden is practically identical to those analysed from Alaska and Greenland.

### Taxonomy

The only species from the Southern Hemisphere which, according to our data belongs in *Massalongia*, is *M. patagonica*, the details of which are as follows:

#### Massalongia patagonica

Taxon classificationFungiPeltigeralesMassalongiaceae

Kitaura & Lorenz in Liu et al.

D7339EEC-797D-544B-B3F3-8DC9C3B78E2F

824006

Massalongia patagonica Kitaura & Lorenz in Liu et al., Sydowia 70: 249–252 (2018) – Holotypus: Argentina, Ushuaia, National Park of Tierra del Fuego, Lapataia Bay, muscicolous on the rock, 54°50'41.42"S, 68°33'52.31"W, 6 m alt., 25 December 2016, *leg.*M.J.Kitaura, J.Bordin, A.A.Spielmann & D.Peralta 4188 (CGMS).

##### Description.

**Figure 3.**
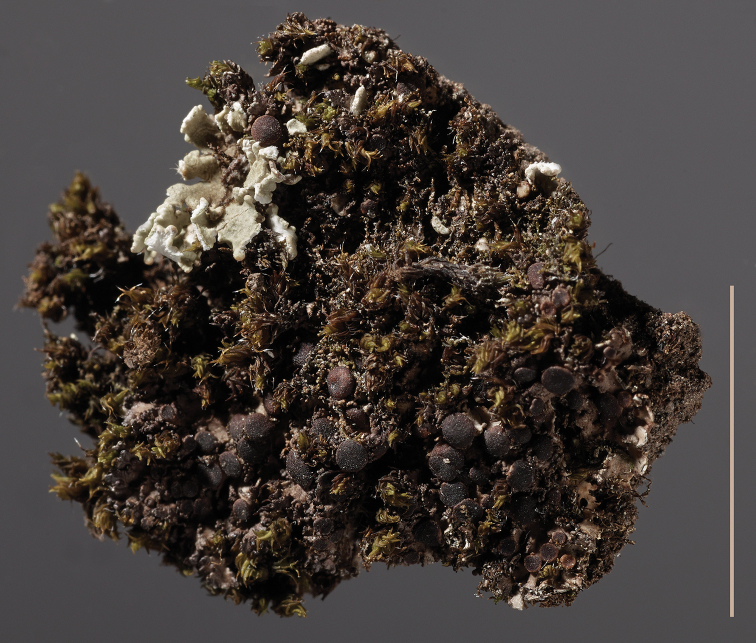
*Massalongia patagonica*, AE 15-033. Scale bar: 1 cm.

**Figure 4.**
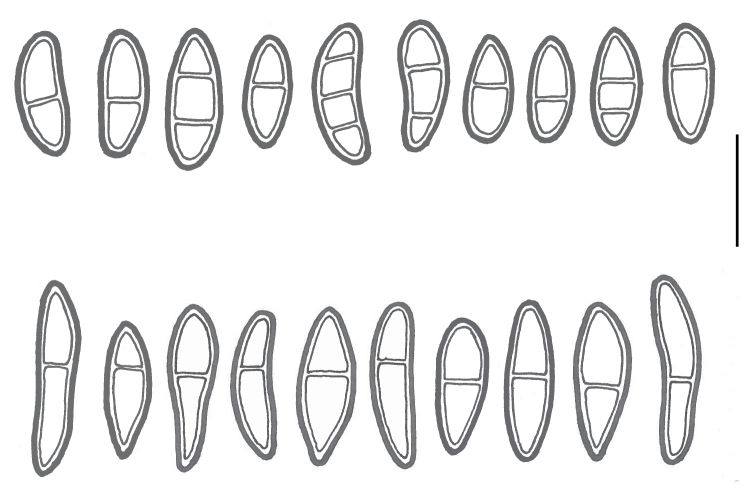
*Massalongia* ascospores, *patagonica* above, *carnosa* below. Scale bar: 20 µm.

*M. patagonica* (Fig. 3) is morphologically similar to *M. carnosa*. Generally, spore characters are the best distinguishing characters (Fig. 4). The spores of *M. carnosa* are longer and 92% of 72 measured spores were in the range 23–35 µm. By contrast, 70% of 58 measured spores of *M. patagonica* were in the range 15–22 µm. This means that there is an overlap in sizes between these two species. However, the spores of *M. patagonica* are often two-septate, sometimes three-septate, which is very rare in *M. carnosa*. Here follows a more detailed treatment of *Massalongia patagonica* Kitaura & Lorenz:

***Thallus*** foliose, forming rosettes up to 3 cm, mostly muscicolous; ***lobes*** 0.5–1.5 mm broad, up to 1 cm long, irregularly and repeatedly divided with isidioid marginal outgrowths, simple to sparingly branched, sometimes developing into branched lobule systems, lobules, 0.1 mm wide. ***Upper surface*** brown, glabrous and glossy; ***upper cortex*** 20–30 µm thick, paraplectenchymatic, of thick-walled (ca. 1.5–2 µm wide) cells with 7–12 µm large lumina; ***photobiont layer*** 40–60 µm thick, often also developed in the subhymenium; ***cyanobiont Nostoc***, cells bluish-green, irregularly subglobose to ellipsoid, 5–9 × 6–11 µm in size, arranged within 20–40 µm large glomeruli without visible chain structures, chain structures visible in some liberated cells; ***medulla*** loose, 60–80 µm thick; ***lower cortex*** absent, with scattered rhizohyphae.

***Apothecia*** common to scattered, substipitate, laminal, 1–2 mm wide; ***thalline excipulum*** lacking, true excipulum weakly prominent; ***epithecium*** 5–10 µm thick, of protruding brown and strongly swollen, pyriform paraphyse end cells, 4–6 µm wide, 7–10 µm long, paraphyses undivided to sparingly divided, 2–4 µm thick; ***hymenium*** ca. 60 µm thick, IKI + blue; ***asci*** clavate 50–70 × 10–15 µm, 8-spored, with distinct internal apical IKI + blue sheath-like structures, sometimes also with weak tube structures; ***ascospores*** narrowly ellipsoid, occasionally asymmetric, 1- to 2 (3)-septate, (13) 20–25 (28) × 5–7.5 µm. ***Hypothecium*** ca. 60 µm thick, weakly brownish, IKI negative. ***Conidiomata*** not seen.

##### Chemistry.

All reactions negative, no lichen substances detected by TLC.

##### Habitat and distribution.

This is a species of wet to dry rock surfaces or boulders, usually growing in between mosses or on plant remains. It has a widely scattered distribution in South America, ranging from the temperate forests of south-central Chile, including the Juan Fernandez Islands and Patagonia, with two widely separated collections from southernmost Chile and Argentinean Tierra del Fuego. In addition, it is known from the Falkland Islands, Antarctica, mountains of SE Australia, where it is rare and from several localities in New Zealand.

##### Specimens examined.

**Antarctica**: South Shetland Islands, King George Isl., Admiralty Bay, creeping slopes above Paradise Cove, 26 Jan 1980, *R. Ochyra* 1224/80 (BG, H); Urbanek, Crag between Polar Committee Glacier and Ladies Icefall, in Ezcurra Inlet, 20 Feb 1980, *R. Ochyra* 2319/80 (BG, H).

**Argentina**: Patagonia: Chubut, Lago Verde, near Futalaufquen, 1 Feb 1950, *I. M. Lamb* 5877 (over mosses on a rock in open scrub, about 30 m above the lake), 5880 (over mosses on a rock in open forest about 15 metres above the lake) (CANL, UPS).

**Australia**: New South Wales, near the summit of Mt. Guthrie, Kosciusko National Park, on moss over granite rocks, 9 Feb 1978, *J.A. Elix* 4360 (CANB); Kosciusko National Park, near Digger’s Creek, 21 Jan 1976, *J. A. Elix* 1722 (CANB); Île Australia near Kerguelen Isl., on moss cushions, 492823S, 695329E, 45 m alt., 31 Dec 2003, *NJM Gremmen*, K-789 (BG).

**Chile**: IX Región de la Araucanía: Reserva Nacional Malalcahuello, W bank of Río Colorado, 500 m W of the Entrance/CONAF building and 200 m S of the junction between the paths Sendero Coloradito and Sendero Sierra de Colorado; 38°25'45"S, 71°32'44"W, 1380 m alt., over *Grimmia* mosses on a S-facing rock outcrop in a *Nothofagus dombeyi-Araucaria araucana* forest, probably affected by river water during high flooding events, 9 Jan 2015, *A. Elvebakk* 15:033 (SGO, BG, UPS, BM, TROM); Archipiélago de Juan Fernandez: Isla Alejandro Selkirk (Mas Afuera), Los Innocentes, 4 Dec 1965, *H. Imshaug* (MSC); Valdivia, Corral, *R. Thaxter* (MSC); XII Región de Magallanes y de la Antárctica Chilena, Provincia Magallanes, Morro Chico, 52°03'S, 71°28'W, 200 m alt., on acrocarpous mosses on a NW-facing rocky slope, 28 Nov 1999, *A. Elvebakk* 99:775 (TROM); Provincia Antártica Chilena, Comuna Cabo de Hornos, Isla Grande de la Tierra del Fuego, Bahía Yendegaia, NNE shore opposite Caleta Ferrari, 54°50'28"S, 68°47'52"W, 13 Jan 2013, *W.R. Buck* 60287 (NY 01886528).

**Falkland Islands**: W. Falkland, Chartres, Luxton NNR, 30 Jan 2015, *A. Fryday* 10999 (MSC).

**New Zealand**: Canterbury, Cass, between Sugar Loaf and Cass Hill, 761 m alt., 18 Feb 1991, *A. J. Fife* 9761 (CHR); Banks Peninsula, Mt. Sinclair, summit, 5 Feb 1970, *D. J. Galloway* (CHR); Mt. Cook National Park, *D. J. Galloway* (CHR); Otago, Deep Stream, above DCC water intake, 13 Feb 1998, *D. J. Galloway* 0170 (CHR); Otago, Old Man Range, N of Obelisk, 5 Feb 2009, *D. J. Galloway* 404009 (CHR); St. Mary’s Range, Anakin’s Skifield, 22 Feb 2006, *D. J. Galloway* (CHR); Lake Onslow near huts, amongst moss in drainage cracks of schist rock in grassland, 30 Jul 1998, *D. J. Galloway* 404012 (CHR); Otago, Pomahaka River- Hukarere, rock slabs above river, 13 Apr 1998, *D.J. Galloway* 404011 (CHR); North Rough Ridge, near “Great Tor”, 12 Apr 1998, *D. J. Galloway* (CHR).

The other taxa originally described from the Southern Hemisphere as *Massalongia* species, are listed alphabetically, according to the epithet at the end of the discussion.

## Discussion

The result of the phylogenetic analyses of *Massalongia* (Fig. 2) show that *Massalongia carnosa* and *M. patagonica* are located in different supported clades, as separate species as also described by Kitaura and Lorenz in Liu et al. (2018), *M. patagonica* being restricted to the Southern Hemisphere, whereas *M. carnosa* occurs only in the Northern Hemisphere. The clade with *M. carnosa* includes one circumarctic and circumboreal species, with low genetic diversity, whereas *M. patagonica* is more variable and shows a geographic pattern within this species. The material from New Zealand groups in a distinct branch within the *M. patagonica* clade and is superficially much more similar to the material of *M. carnosa*, but has extra short ascospores measured in two samples from New Zealand, all spores were shorter than 23 µm. This could be a result of the preference for moist, mossy habitats (Galloway 2007) as opposed to the drier, often exposed habitats in Chile. The material from Australia and New Zealand is, therefore, best classified as part of the *M. patagonica* complex.

That species is also found as far west as the Juan Fernandez Islands and is also possibly present on the Antarctic Peninsula and the Bouvet Island, but the material examined was sparse, sterile and too old for molecular studies.

Still, *M. patagonica* is not morphologically easily distinguished from *M. carnosa*; the two species have different spores, although there is an overlap zone in both length and degree of septation. Both species have a gross morphology showing high variation, probably due to habitat modifications, depending on light exposure, competition, moisture and water availability.

Chilean material of *M. patagonica* tends to have thicker, narrower and clearly radiating lobes than most material of *M. carnosa*. However, in cases where habitat information is available, they appear to be dry, but exposed to nutrient supplies by spring flooding (the Río Colorado collection), wind-transported saline lake dust (the Morro Chico collection) or seashore spray (the Tierra del Fuego collection). The New Zealand material, on the other hand, treated as *M. carnosa*, is cited as widespread and from moist habitats by Galloway (2007).

This detailed phylogenetic signal within *M. patagonica* is the result of a long history of evolution and isolation in austral areas, although shorter than the split-up between *M. carnosa* and *M. patagonica*. There is a record of *M. carnosa* from Mt. Kinabalu on Borneo (Sipman 1993) which could have indicated a migration route between a northern and a southern distribution area of the genus; however, a check of the material deposited at herbarium B revealed that it instead represents a sterile, richly squamulose specimen of a *Parmeliella* species. Future studies should investigate phylogeographic relationships between the three accepted species and the molecular distances between *M. patagonica* in New Zealand, Australia and South America/West Antarctica.

The examination of all relevant material from the Southern Hemisphere, shows the following, treated alphabetically according to the epithet:

***Massalongia antarctica*** Dodge is a species only known from the type specimen from Lambda Island at the tip of the Antarctic Peninsula (Siple 380c-2, FH!). The type specimen is minute and sterile and consists of two different species, none of which belongs in *Massalongia*. The one fitting best with the description has a crustose, hemi-gelatinous thallus in accordance with species of the Arctomiaceae. There are no apothecia present in the collection and the description of the apothecia, given by Dodge (1968), is at variance with characters of *Massalongia*, indicating a species of the Arctomiaceae, most probably in *Arctomia*. There is, however, no known species with such a distinctly crustose thallus. More material is needed to identify this taxon more exactly. The sample also contains squamules with a trebouxioid photobiont and this is possibly *Pertusaria corallifera* Vain. as pointed out by Castello and Nimis (1995).

***Massalongia griseolobata*** Øvstedal is a species only known from the type specimen (from Gough Isl., coll. Gremmen 2006-91, BG!). Even if only incipient apothecia were found, we do not hesitate to place this species in the Pannariaceae, based on morphology and the original description of the asci. They are recorded to have apically blue in tholus in iodine with a weak ring-structure. (*Massalongia* has sheet-like structures, Jørgensen 2007). The molecular study confirms this (Fig. 1). The species groups in the parmelielloid clade (Clade 1) in the tree by Ekman et al. (2014), with *Degeliella*, *Leiderma*, *Erioderma* and *Joergensiana*. This is an unresolved group of subantarctic taxa (Jørgensen and Andersen 2015) in need of further studies.

***Massalongia intricata*** Øvstedal was correctly transferred to the genus *Steinera* by Ertz et al. (2017). *S. intricata* has a semi-gelatinous thallus producing apothecia on special lobules, just as species in the Arctomiaceae.

***Massalongia novozelandica*** Dodge was recorded by Galloway (2007), but the holotype (the only material) has not been possible to obtain. However, the original description of the spores being brownish at maturity with disappearing septae (clearly pseudoseptae) is at variance with characters found in *Massalongia*. We agree with Galloway that this is probably a parasite growing on the thallus of a species in the Pannariceae.

***Massalongia olechiana*** Alstrup and Søcht. was correctly transferred to the genus *Steinera* in the Arctomiaceae by Ertz et al. (2017).

***Massalongia patagonica*** Kitaura and Lorenz, the recently described species (Fig. 3), belongs in the genus and, according to our phylogenetic tree (Figs 1, 2), prove to be distinct from *M. carnosa*, the latter being restricted to the Northern Hemisphere. The two species are morphologically variable due to the ecological conditions, but have different spores (Fig. 4), usually shorter than 25 µm in *M. patagonica*, but variable both in length and number of septae in both species. Chilean material of *M. patagonica* tends to have thicker, narrower and clearly radiating lobes than most material of *M. carnosa.*

## Conclusion

From these facts, we conclude that there is only one, widespread species of *Massalongia* in the Southern Hemisphere, *M. patagonica*, though the populations in Australia and New Zealand differ somewhat molecularly, but more data is necessary to decide their taxonomic status. *M. patagonica* has a wider distribution than indicated in the original paper, also southwards and westwards. Previous records of several species in the Southern Hemisphere proved incorrect, most of them belonging in other genera.

The type species *M. carnosa* is restricted to the Northern Hemisphere, where it is widespread and variable, but without distinct molecular groupings requiring taxonomic recognition. There is also a local endemic, *M. microphyllizans* in California.

## Supplementary Material

XML Treatment for Massalongia patagonica
